# Can suppressive antibiotics be avoided in the postnatal management of antenatally detected obstructive hydronephrosis?

**Published:** 2007

**Authors:** Sreedhar V. Reddy, Arun Chawla, Joseph Thomas

**Affiliations:** Department of Urology, Kasturba Medical College, Manipal, India. E-mail: urologyreddy@yahoo.com

## SUMMARY

This paper retrospectively evaluated the role of prophylactic antibiotics in the postnatal management of antenatally detected obstructive hydronephrosis. 105 neonates (82 males and 23 females) with grade III or IV fetal hydronephrosis were followed up between 1994 and 2004. Hydronephrosis was graded by the Society of Fetal Urology (SFU) ultrasound grading system. The study also tried to correlate the chances of getting urinary tract infection (UTI) and the level of upper urinary obstruction. None of the patients in this study received prophylactic antibiotics. All the 82 males were not circumcised. 99 neonates in the study had unilateral and 6 had bilateral hydronephrosis. All the patients had an obstructive pattern in the diuretic renogram with half time of 20 minutes or more. 75 patients had ureteropelvic junction obstruction (UPJO) and 30 had lower urinary obstruction (LUO). The clinical course of the patients was reviewed for 12 months. In those neonates who developed UTI, corrective surgery was performed.

The overall incidence of UTI during the follow up period was 36.2% (38 patients). Majority of the cases occurred before 6 months of age (92.8%). The mean age of onset of UTI was 2.6 months. There was a significant difference noted in the incidence of UTI and the level of obstruction. While UTI developed in 50% of patients with LUO, only 30.7% of patients with UPJO developed UTI. In the LUO group, the incidence of UTI was 70% in those with ureterocele, 50% in those with ectopic ureter and 38.9% in those with ureterovesical junction obstruction. 77 neonates (73.3%) underwent corrective surgery at a mean age of 3.8 months. The incidence of UTI before surgical correction was 33.8% at a mean age of 2.1 months. The multivariate analysis showed that the level of obstruction in the operated cases was the only significant variable in the development of UTI - 54.2% for LUO vs. 24.5% for UPJO. 12 of the 28 patients (42.9%) followed up without surgery developed UTI. Of the 6 patients with bilateral UPJO, 2 (33.3%) developed UTI.

## COMMENTS

Hydronephrosis is the most common genitourinary tract anomaly identified on prenatal ultrasound studies. Ureteropelvic junction obstruction accounts for approximately 50% of the cases of prenatally detected hydronephrosis.[1,2] Hydronephrosis in the newborn period needs to be managed differently because these kidneys are physiologically different. The increased awareness of this aspect of the developing kidney has shifted the postnatal management of antenatally detected hydronephrosis from an aggressive surgical to a more conservative approach.[3,4] Conservative approach has become the standard for unilateral hydronephrosis. It has been advocated for bilateral hydronephrosis also.[5,6] The possibility of UTI developing in the obstructed system during the conservative period has made antibiotic prophylaxis an integral part of the observational management.[1,4] However there are no unequivocal data which showed that antibiotic prophylaxis was beneficial and cost effective for all congenital hydronephrosis. The important question that needs to be answered is whether avoiding antibiotic prophylaxis in the neonatal period would increase the risk of UTI. The incidence of UTI was not increased in those studies with PUJO where antibiotic prophylaxis was not given.[5,7] However antibiotics are required in the conservative management of LUO with ureterocele or megaureter as there was a higher incidence of UTI in these conditions.[8]

The retrospective design of the present study and the lack of control groups are major flaws in arriving at scientific conclusions. However from the present study it is worth noting that, there was a higher incidence of UTI in children when antibiotics were not used. As the time of onset of UTI was within the first 6 months, it is suggested that antibiotics should be given from birth. The higher incidence of UTI in this study could be due to the inclusion of patients with SFU grade III and IV and exclusion of SFU grade I or II from the study. Another interesting aspect from this study was the higher incidence of UTI with lower tract obstruction compared to ureteropelvic junction obstruction. The authors suggested that the greater volume of retained urine in LUO and the associated bladder dysfunction must have predisposed to infection. However there was no convincing evidence to substantiate this in this study.
